# Adverse Events as a Potential Clinical Marker of Antitumor Efficacy in Ovarian Cancer Patients Treated With Poly ADP-Ribose Polymerase Inhibitor

**DOI:** 10.3389/fonc.2021.724620

**Published:** 2021-09-06

**Authors:** Jing Ni, Xianzhong Cheng, Rui Zhou, Qian Zhao, Xia Xu, Wenwen Guo, Hongyuan Gu, Chen Chen, Xiaoxiang Chen

**Affiliations:** ^1^Department of Gynecologic Oncology, The Affiliated Cancer Hospital of Nanjing Medical University, Jiangsu Cancer Hospital, Jiangsu Institute of Cancer Research, Nanjing, China; ^2^Department of Chemotherapy, The Affiliated Cancer Hospital of Nanjing Medical University, Jiangsu Cancer Hospital, Jiangsu Institute of Cancer Research, Nanjing, China; ^3^Department of Pathology, The Second Affiliated Hospital of Nanjing Medical University, Nanjing, China

**Keywords:** PARP inhibitor, ovarian cancer, efficacy, clinical marker, adverse events

## Abstract

**Background:**

PARP inhibitor (PARPi) is an important progress in ovarian cancer treatment. The available evidence suggests that BRCA mutation and homologous recombination deficiency (HRD) are effective biological markers for PARPi. Here we investigated the relationship between adverse events (AEs) and efficacy of PARPi in ovarian cancer patients.

**Methods:**

Seventy-eight patients with ovarian cancer patients underwent Olaparib and Niraparib from July 2018 to July 2020 were analyzed. AEs were assessed by the National Cancer Institute Common Terminology Criteria for Adverse Events (NCI CTCAE) v5.0. Chi-square test or fisher exact tests was performed to observe the association between categorical variables. Logistic regression analysis was conducted to investigate the independent variables for disease control response (DCR). Progression-free survival (PFS) was compared between AEs variables by log-rank test.

**Results:**

Patients with AEs in the first one week had a higher DCR compared with those after one week (86.11% *versus* 60.98%, p=0.013). Patients with serious AEs (SAEs) had a significantly higher DCR (81.40% *versus* 60.60%, p=0.045). There were associations between anemia and DCR in both occurrence (79.63% *versus* 56.52%, p=0.037) and grade (100% *versus* 73.17%, p=0.048). The median PFS of patients with hematological toxicity was longer than that of patients with no-hematological toxicity (30 *versus* 20 weeks, p=0.047). Patients with hematological toxicity within four weeks had prolonged median PFS than those with hematological toxicity after four weeks (40 *versus* 22 weeks, p=0.003).

**Conclusions:**

The early presence of AEs and SAEs in hematological toxicity of PARPi were related to the antitumor efficacy, which might be a valid and easily measurable clinical marker in ovarian cancer patients.

## Introduction

Ovarian cancer remains the first leading cause of cancer death in gynecological malignancy (1). Seventy percent of ovarian cancer patients can benefit from the traditional standard first-line treatment including cytoreductive surgery followed by platinum-based chemotherapy (2). However, about 80% patients will develop disease recurrence after traditional initial treatment and ultimately progress to platinum-resistance ovarian cancer (3). Recently, PARPi have transformed the treatment landscape of patients with ovarian cancer (4–11).

DNA damage in cells manifests mainly as single-strand breaks (SSBs), double strand breaks (DSBs) or replication fork stalling (12). PARP1 and PARP2 enzymes play an important role in the repair of SSBs in DNA, and they can recognize and bind to the DNA fracture site, and mediate DNA single-strand damage repair in tumor cells. HRD-positive tumor cells (cells with BRCA mutation or other mutations in homologous recombination repair (HRR) pathway genes such as RAD51 and ATM) cannot repair DNA single-strand damage, forming the synthetic lethal effect (13). Therefore, BRCAmt or HRD-positive tumor cells are more sensitive to PARPi in terms of molecular mechanisms (14).

PARPi are recommended as maintenance treatment and multi-line treatment in ovarian cancer patients according to National Comprehensive Cancer Network (NCCN) guidelines. The five-year follow-up data of SOLO1 showed that nearly 50% of patients harbored BRCA mutation have not progress with olaparib as first-line maintenance treatment, compared with 20% of patients in placebo group in 2020 ESMO meeting (15). Furthermore, olaparib as second-line maintenance treatment significantly increased progression-free survival (PFS) and overall survival (OS) for patients with BRCA mutation in SOLO2 study (16, 17). Both NOVA and PRIMA studies demonstrated that patients with HRD positive could get more benefit from niraparib as maintenance treatment (4, 5). On the other hand, olaparib could be used as single-agent therapy for multi-line treatment in ovarian cancer patients harbored BRCA mutation (18). QUADRA study demonstrated that women with heavily pretreated ovarian cancer, especially in patients with HRD positive platinum-sensitive disease, which included not only patients with BRCA mutation but also population with BRCA wild-type could benefit from niraparib (19). Previous clinical trials showed that ovarian cancer patients with BRCA mutation or HRD positive were more likely to benefit from PARPi. It was confirmed that BRCA mutation or HRD positive was an effective predictive biomarker for efficacy of PARPi from both molecular mechanisms and clinical practice.

However, there were no early clinical biomarkers to predict the efficacy. We observed that most patients suffered different PARPi-related adverse events (PrAEs) that might correlate with prognosis in our previous real-world studies (20). Based on these observations, we conducted this study to investigate the association of PrAEs with clinical outcomes in ovarian cancer patients.

## Materials and Methods

### Study Population

Between July 2018 to July 2020, seventy-eight advanced ovarian cancer/fallopian tube cancer/peritoneal cancer patients treated with PARPi, including olaparib with initial dose as 300 mg twice-daily and niraparib with initial dose as 200mg once-daily that was based on the baseline weight or platelets were enrolled in Jiangsu Cancer Hospital. If the patient experienced SAEs (Grade 3-4), the dose reduction and interruption would be done according to drug instruction of olaparib or niraparib. Treatment discontinued until the occurrence of radiological progression, as defined by Response Evaluation Criteria in Solid Tumors 1.1 (RECIST 1.1), unacceptable adverse events or death. Basic characteristics were collected from these patients. Platinum-sensitive ovarian cancer was defined as patients who relapsed more than or equal to 6 months after initial treatment and platinum-resistant ovarian cancer was considered as patients who progressed during initial treatment, or relapsed less than 6 months after initial treatment.

The inclusion criteria for all patients included histologically confirmed advanced ovarian cancer, fallopian tube cancer, peritoneal cancer, taking PARPi for more than four weeks, at least one measurable lesion as defined by RECIST 1.1, an Eastern Cooperative Oncology Group performance status (ECOG PS) of 0 or 1, and acceptable hematologic, hepatic, and renal function. Patients were excluded if they received platelet or red blood cell infusion within 4 weeks before taking the drug and had other malignant diseases within 2 years. All methods were performed in accordance with the relevant guidelines and regulations by the ethics committee of Jiangsu Cancer Hospital (2020- science-040).

### Assessments

Patient demographics, adverse events and treatment efficacy were available and collected from all enrolled subjects. Efficacy assessments were performed based on computed tomography at baseline, after two and three cycles, and every 8 weeks thereafter until disease progression. The baseline of serum CA125 and a following monthly examination of CA125 were also conducted. The efficacy was assessed as complete response (CR), partial response (PR), stable disease (SD) and progressive disease (PD) by RECIST 1.1. Disease control rate (DCR) was defined as the proportion of patients achieving CR, PR or SD for at least 12 weeks. PFS was assessed from the first day of treatment with PARPi to disease progression or death from any cause. Treatment-related AEs were graded according to NCI CTCAE 5.0.

### Statistical Analysis

Data were statistically analyzed using SPSS version 19.0 professional statistical software and all the count data were expressed as a percentage (%). Baseline characteristics and AEs were compared using t tests for continuous variables and fisher’s exact or chi-squared tests for categorical variables. Logistic regression analysis was conducted to investigate the association between independent variables and DCR. PFS was assessed using Kaplan–Meier method and compared between AEs variables by log-rank test. Single factors with p < 0.10 were defined as independent variable. Multivariate cox regression analysis was conducted to investigate the association between independent variables and PFS. A two-sided p-value less than 0.05 was considered statistically significant.

## Results

### Patient Characteristics

The demographic and baseline characteristics of the seventy-eight patients were summarized in **Table S1**, of whom seventy-four patients were ovary cancer and four patients were fallopian tube cancer. The median age of patients was 56 years (range 30–80 years). The median follow-up time was 22 weeks (range 12–88 weeks), and median PFS was 28 weeks with 95% confidence interval (CI) of 21.6–34.4%. Among overall population, the overall DCR was 72.7% (95% CI: 62.6–82.9%) and ORR was 14.3% (95% CI: 6.3–22.3%).

Of those, forty-eight patients (61.5%) treated with olaparib and the remaining thirty patients (38.5%) treated with niraparib. During olaparib treatment, a total of thirty-seven patients experienced anemia, twelve of whom were diagnosed with grade 3-4 anemia. Thrombocytopenia occurred in seven patients, two of whom were grade 3-4. In patients treated with niraparib, seventeen patients had mild (grade 1–2) anemia except for one case with grade 4 anemia. Thrombocytopenia developed in eighteen patients, six of whom had grade 3-4 thrombocytopenia. In addition, there were 83.1% patients suffered fatigue, 66.2% patients had nausea, and 62.3% patients experienced decreased appetite in total subjects.

### AEs and DCR

This cohort analysis showed that early presence of AEs (within one week), SAEs, residual disease at initial surgery, and ECOG ps were associated with DCR. Patients with AEs in the first one week had a higher DCR compared with after one week (86.11% *versus* 60.98%, p=0.013). Also patients with SAEs had a significantly higher DCR (81.40% *versus* 60.60%, p=0.045). Besides, the DCR among patients with R0 resection was higher (83.33% *versus* 54.84%, p=0.008) than those with R1 resection. The same results were also observed in patients with ECOG 0 (83.33% *versus* 60.00%, p=0.038) compared with those with ECOG1 (**Table 1**).

**Table 1:** Disease control rate of patients with different baseline characteristics and adverse events.

Baseline characteristics	Disease control number	DCR (%)	*χ* ^2^	*P*-value	AEs	DCR (%)	*χ* ^2^	*P*-value
Time before initial AEs occurred			6.106	0.013	Hematological adverse events		1.322	0.250
≤1 week	31	86.11			Yes	76.67		
>1 week	25	60.98			No	58.82		
Residual disease at PDS/IDS			7.067	0.008	Time before hematological toxicity occurred		3.297	0.069
R0	35	83.33			≤4 weeks	84.21		
R1	17	54.84			>4 weeks	63.64		
ECOG PS			5.240	0.038	Anemia		4.342	0.037
0	35	83.33			Yes	79.63		
1	21	60.00			No	56.52		
SAEs (grade 3-4)			4.035	0.045	Time before anemia occurred		0.024	0.878
Yes	35	81.40			≤4 weeks	81.82		
No	20	60.60			>4 weeks	76.19		
Categories of PARP inhibitors			0.002	0.962	Thrombocytopenia		0.417	0.518
Olaparib	35	72.92			Yes	68.00		
Niraparib	21	72.41			No	75.00		
Neoadjuvant chemotherapy			0.728	0.394	Neutropenia		0.007	0.933
Yes	9	60.00			Yes	76.47		
No	40	75.47			No	71.67		
Multi-line chemotherapy			1.660	0.198	Nausea		1.305	0.253
Yes	25	65.79			Yes	64.00		
No	27	79.41			No	76.47		
PDS/IDS			0.464	0.496	Fatigue		0.232	0.630
Yes	52	71.23			Yes	66.67		
No	4	100.00			No	73.44		
Secondary cytoreductive surgery			0.005	0.941	Decreased appetite		0.451	0.502
Yes	11	68.75			Yes	67.86		
No	42	73.68			No	75.00		
Family history of cancer			0.000	1.000	Grade of anemia		Exact probability test	0.048
Yes	10	66.67			1-2	73.17		
No	20	71.43			3-4	100.00		
HRD status			0.928	0.355	–		–	–
Positive	25	71.43			–	–		
Negative	8	57.14			–	–		
Age, years			0.468	0.494	–		–	–
≤55	24	68.57			–	–		
>55	31	75.61			–	–		
International FIGO stage			2.215	0.137	–		–	–
≤IIIa	20	83.33			–	–		
>IIIa	32	66.67			–	–		

DCR, Disease control rate; AEs, adverse events; PDS, primary debulking surgery; IDS, interval debulking surgery; R0, no macroscopic disease; R1, 1 cm or less; ECOG PS, Eastern Cooperative Oncology Group performance status; SAEs, serious AEs; HRD, homologous recombination deficiency; FIGO, International Federation of Gynecology and Obstetrics.

In the further analysis of each AE, it was found that there were relationships between anemia and DCR in both occurrence (79.63% *versus* 56.52%, p=0.037) and grade (100% *versus* 73.17%, p=0.048) (**Table 1**). Similarly, DCR among patients treated with olaparib was association with occurrence of anemia (83.78% *versus* 36.36%, p=0.007). However, patients treated with niraparib had a higher DCR in those experienced thrombocytopenia within four weeks than after four weeks (86.67% *versus* 40.00%, p=0.044) (**Table S2**). Baseline characteristics between the occurrence of AEs were not significantly different (**Table S3**).

A multivariable logistic regression model was constructed to predict DCR in the study population. It showed that the occurrence of AEs (odds ratio (OR): 0.162; 95% CI: 0.041-0.643, p = 0.010), ECOG score (OR: 0.188; 95% CI: 0.051-0.684, p=0.011) and residual disease at initial surgery (OR: 0.275; 95% CI: 0.084-0.903, p=0.033) were statistically significant for predicting DCR (**Table 2**). After internal verification of the existing population by logistic model, it was found that the accuracy rate of three factors including occurrence of AEs, ECOG score and residual disease at initial surgery for DCR was 96.1% and the total accuracy for DCR or PD was 76.4% (**Table S4**).

**Table 2:** Logistic regression analysis (Forward: LR) of multi-factor for predicting disease control rate.

	B	SE	Wald	df	*P*-value	OR	95% CI of OR	
							Lower	Upper
Residual disease at PDS/IDS	-1.292	0.607	4.527	1	0.033	0.275	0.084	0.903
ECOG PS	-1.673	0.660	6.426	1	0.011	0.188	0.051	0.684
Time before initial AEs occurred	-1.819	0.703	6.704	1	0.010	0.162	0.041	0.643

B, regression coefficient; SE, standard error; df, degree of freedom; OR, odds ratio; CI, confidential interval; PDS, primary debulking surgery; IDS, interval debulking surgery; ECOG PS, Eastern Cooperative Oncology Group performance status; AEs, adverse events.

### AEs and PFS

**Figure 1:**
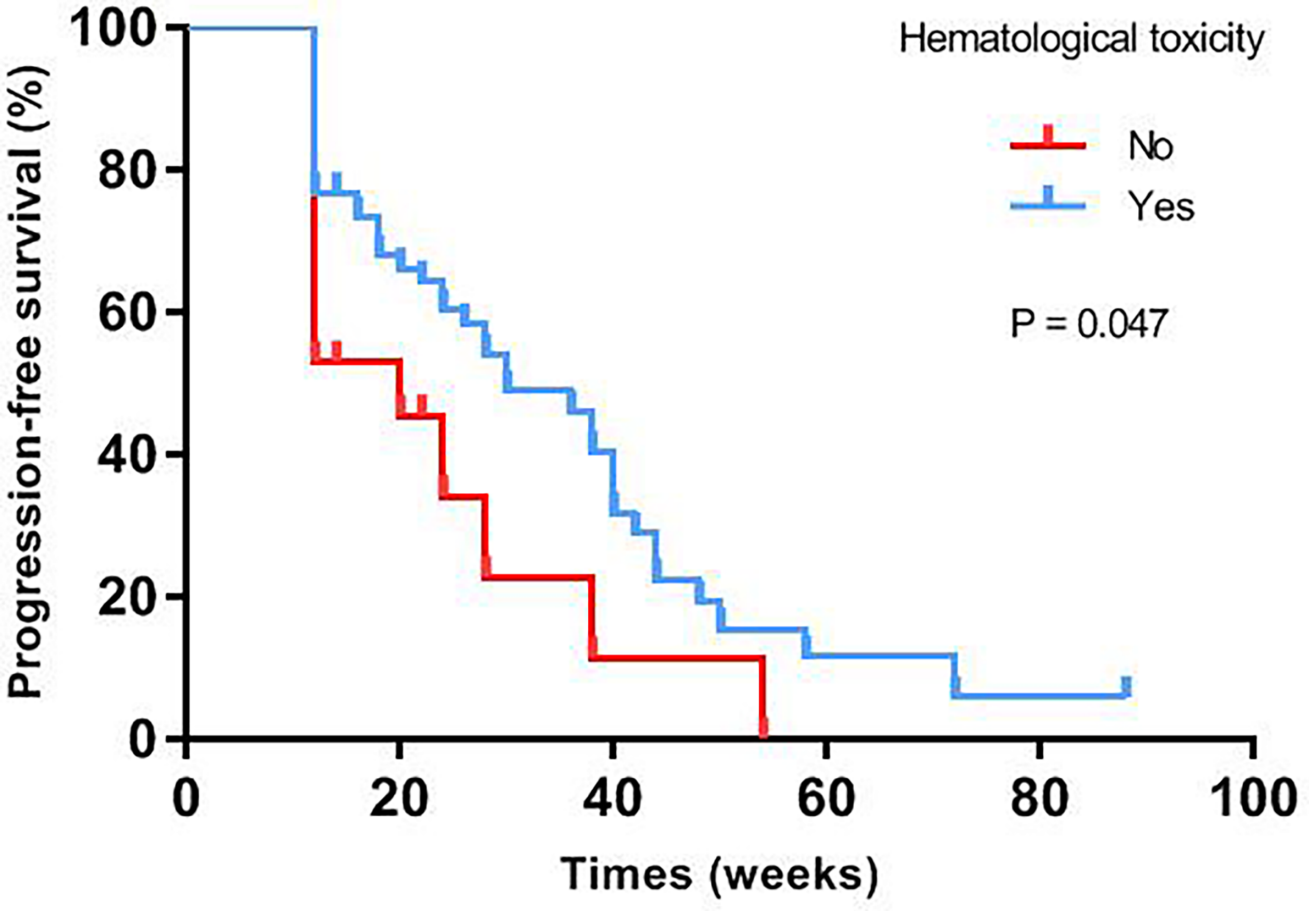
PFS was compared between patients with or without hematological toxicity.

**Figure 2:**
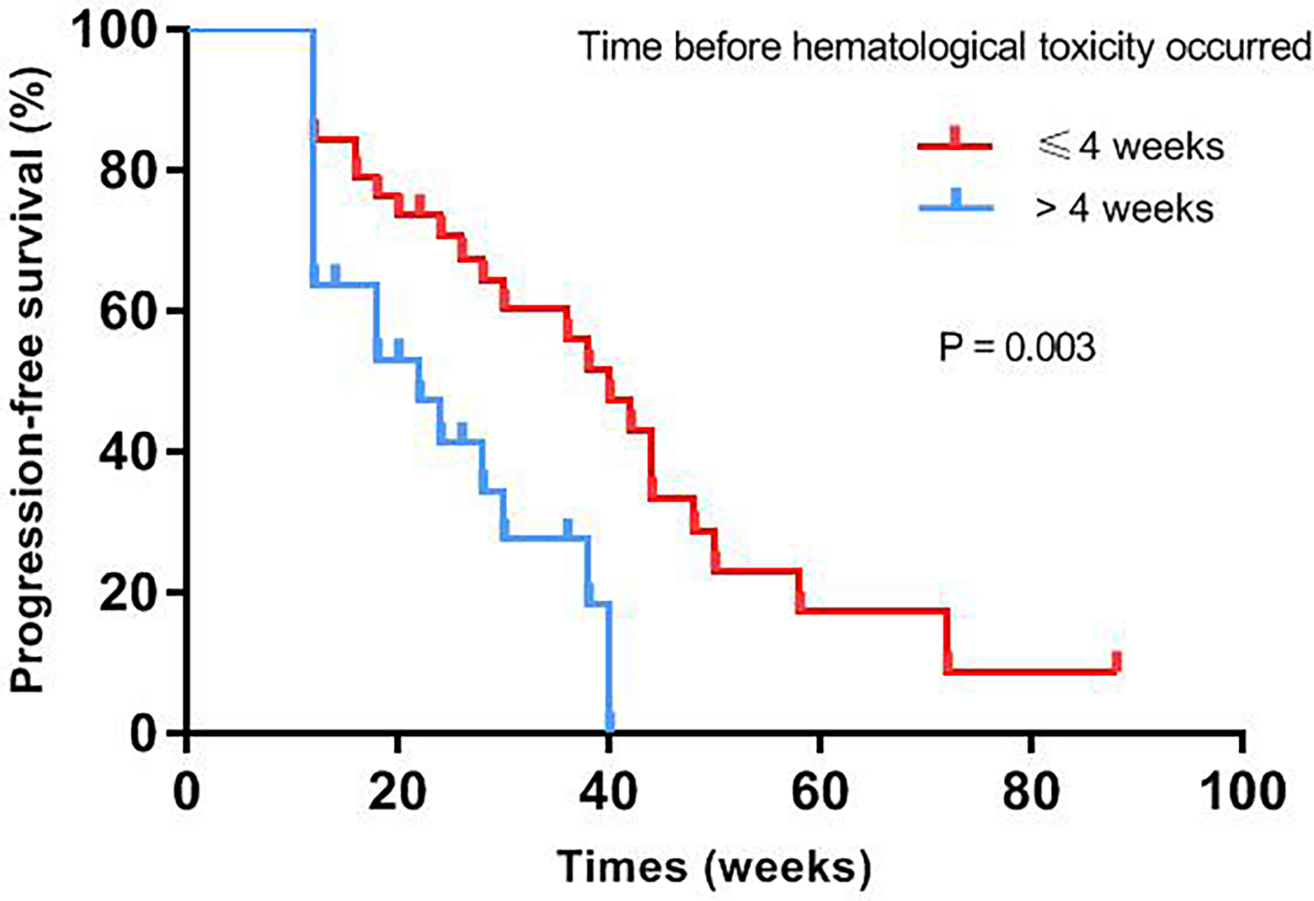
PFS was compared between patients with hematological toxicity within 4 weeks or after 4 weeks.

Median progression-free survival of patients with different baseline characteristics and adverse events were presented in **Table 3**. Univariate log-rank test analysis showed that the PFS among patients with hematological toxicity was longer (median: 30 weeks [95% CI: 20.78, 39.22]) than with no-hematological toxicity patients (median: 20 weeks [95% CI: 13.61, 26.39], p=0.047) (**Figure 1**). Patients with hematological toxicity within four weeks had prolonged median PFS than who with hematological toxicity after four weeks (40 *versus* 22 weeks, p=0.003) (**Figure 2**). Multivariate Cox regression analysis showed that hematological toxicity after four weeks (HR: 2.613; 95% CI:1.104-6.187, p=0.029), residual disease at PDS/IDS(R1/R2) (HR: 3.579; 95% CI:1.443-8.880, p=0.006) and BRCAmt (HR:0.301; 95% CI:0.123-0.739, p=0.009) were the independent factors (**Table 4**). Further interaction analysis with these independent factors found that there was no interaction between BRCAmt and hematological toxicity within four weeks [(relative excess risk due to interaction, (RERI): -1.246; 95% CI: -4.255-1.763], residual disease at PDS/IDS and hematological toxicity within four weeks (RERI: 2.134; 95% CI: - 3.270-7.538).

**Table 3:** Median progression-free survival of patients with different baseline characteristics and adverse events.

Baseline Characteristics	Median PFS (weeks)	Logrank test *χ*^2^	*P*-value	AEs	Median PFS (weeks)	Log rank test *χ*^2^	*P*-value
Residual disease at PDS/IDS		3.573	0.059	Hematological adverse events		3.933	0.047
R0	38			Yes	30		
R1	22			No	20		
ECOG PS		0.843	0.358	Time before hematological toxicity occurred		8.961	0.003
0	36			≤4 weeks	40		
1	28			>4 weeks	22		
SAEs (grade 3-4)		0.036	0.850	Anemia		1.928	0.165
Yes	28			Yes	36		
No	30			No	20		
Categories of PARP inhibitors		1.707	0.191	Time before anemia occurred		3.221	0.073
Olaparib	30			≤4 weeks	38		
Niraparib	24			>4 weeks	28		
Neoadjuvant chemotherapy		0.208	0.648	Thrombocytopenia		0.674	0.412
Yes	28			Yes	24		
No	30			No	30		
Multi-line chemotherapy		1.884	0.170	Neutropenia		2.564	0.109
Yes	24			Yes	40		
No	38			No	26		
PDS/IDS		0.023	0.879	Nausea		1.109	0.295
Yes	28			Yes	30		
No	24			No	18		
Secondary cytoreductive surgery		1.199	0.274	Fatigue		0.219	0.639
Yes	28			Yes	28		
No	38			No	24		
Family history of cancer		0.002	0.964	Decreased appetite		0.356	0.551
Yes	30			Yes	24		
No	28			No	30		
HRD status		2.212	0.137	Grade of anemia		1.795	0.180
Positive	36			1-2	30		
Negative	12			3-4	40		
BRCA status		3.338	0.068				
Positive	18						
Negative	36						
Age, years		0.421	0.516				
≤55	28						
>55	24						
International FIGO stage		2.021	0.155				
≤IIIa	36						
>IIIa	28						

PFS, progression-free survival; PDS, primary debulking surgery; IDS, interval debulking surgery; R0, no macroscopic disease; R1, 1 cm or less; ECOG PS, Eastern Cooperative Oncology Group performance status; SAEs, serious AEs; HRD, homologous recombination deficiency; FIGO, International Federation of Gynecology and Obstetrics; AEs, adverse events.

**Table 4:** Cox regression analysis of multi-factor for predicting progression-free survival.

	B	SE	Wald	df	*P*-value	HR	95% CI of HR	
							Lower	Upper
Time of HT occurred	0.961	0.440	4.773	1	0.029	2.613	1.104	6.187
Residual disease at PDS/IDS	1.275	0.464	7.566	1	0.006	3.579	1.443	8.880
BRCA	-1.199	0.457	6.878	1	0.009	0.301	0.123	0.739

Single factors with p < 0.10 were defined as independent variable.

B, regression coefficient; SE, standard error; df, degree of freedom; HR, hazard ratio.

## Discussion

PARPi is an important milestone in ovarian cancer treatment. Clinical studies showed that most patients experienced different degrees of AEs after taking PARPi. And mild or moderate AEs, namely CTCAE grade 1-2 is more common, including hematologic toxicity, gastrointestinal reactions and fatigue. Most of the hematologic laboratory abnormalities occurred within the first three months. The incidence of grade 3 or 4 anemia, thrombocytopenia and neutropenia was the main reason of dose reduction, interruption and even discontinuation. 10-15% of patients terminated their medication due to adverse reactions, and most patients could be treated with long-term medication (4, 5).

Similar adverse events were also observed in our previous real-world studies as well as in the population of this study. Interesting, it was observed that AEs of PARP inhibitors were highly similar to the traditional cytotoxic drugs that might be related to the distribution of PARP in various tissues of the body (20, 21). And molecular mechanism of PARP inhibitors is different from traditional targeted drugs which are targeted at a known oncogenic site, whether a protein molecule or a gene fragment. PARP inhibitors have high therapeutic index and low off-target effect based on the mechanism (22). Therefore, we combined with our clinical observation and mechanism of PARPi to further speculated that the AEs of PARP inhibitors might be related to the efficacy.

Some studies have found that there is a correlation between AEs of apatinib and the efficacy in treatment of gastric cancer, non-small cell lung cancer, colorectal cancer and liver cancer that may be due to the simultaneous expression of vascular endothelial growth factor (VEGF) and platelet-derived growth factor (PDGF) receptors in tumor tissues and normal tissues (23–27). Recent studies reported that patients who experienced immune-related adverse events demonstrated marked improvements in survival and response rate compared to those lacking toxicity, which might be triggered by antigens that were common to both tumor and inflamed organ (28–30). Similar to the mechanism of immune-related adverse events, the correlation in PARP inhibitors is likely to be related to the widespread distribution of PARP in the cells. At present, there are no studies on the efficacy and AEs of PARPi.

In this study, we found that early presence of AEs (within one week), SAEs, residual disease at initial surgery, and ECOG score were correlated with the short efficacy of PARPi. But multivariable analysis showed that early presence of AEs, ECOG score and residual disease at initial surgery were statistically significant for DCR. The accuracy rate of these three factors for DCR was 96.1% and the total accuracy for DCR or PD was 76.4% through internal verification of the existing population which needed to be further performed by external validation. DCR among patients treated with olaparib and niraparib were association with anemia and thrombocytopenia, respectively. However, all patients who experienced anemia had a higher DCR that might be attributed to more enrolled patients taking olaparib. In addition, the prolonged PFS was observed among patients with hematological toxicity and hematological toxicity within four weeks, especially the latter. Further interaction analysis verified that hematological toxicity within four weeks, residual disease with R0 at PDS/IDS and BRCAmt were three independent factors for the efficacy of PARPi. The differences between PFS and DCR related factors were due to the small sample and short follow-up time.

Small sample size and diverse cohort is the most critical limitation in our single-center analysis that may affect parts of results to demonstrate statistically significant differences. The data of overall survival were lacking in our study because PARPi was approved in China not long ago. The level of evidence for our retrospective study was insufficient. In clinical practice, it may only be used in the process of patients using PARP inhibitor to roughly evaluate the immediate or short-term efficacy. RECIST 1.1 is still the evaluation standard of curative effect. And further randomized studies should be performed to evaluate the role of PrAEs as a potential prognostic marker in advanced ovarian cancer patients treated with PARPi. Therefore, we recently initiated a prospective study to that intended to confirm the results of this retrospective study, and to further explore other possible clinical markers and the possibility of establishing a comprehensive evaluation model for the efficacy of PARP inhibitors (Clinical trial information: NCT04582552).

In conclusion, we firstly found that the early presence of AEs, and SAEs in hematological toxicity of PARPi were related to the antitumor efficacy, which might be a valid and easily measurable clinical marker in ovarian cancer patients.

## Data Availability Statement

The datasets presented in this article are not readily available because we would not share the data and material used in this article, because we need them for further research. Although it is available from the corresponding author on reasonable request. Requests to access the datasets should be directed to cxxxxcyd@gmail.com.

## Ethics Statement

The studies involving human participants were reviewed and approved by Jiangsu Cancer Hospital’s Ethical Committee. The patients/participants provided their written informed consent to participate in this study.

## Author Contributions

JN participated in the design of present study and drafted the manuscript. XianC and RZ carried out the cases recruit of present study. QZ and HG participated in the cases recruit of present study. WG carried out statistical analysis. XX and CC participated in the statistical analysis and drafted the manuscript. XiaoC designed of the study, performed the statistical analysis and revised the manuscript. All authors contributed to the article and approved the submitted version.

## Funding

This study was supported by grants from the National Natural Science Foundation of China (No. 81472441, 81501205), Jiangsu Provincial Scientific research and Health Project for Women and Children (No. F202004), Natural Science Foundation of Youth Fund Projects of Jiangsu Province (SBK2021040731), Institute level project of Jiangsu Cancer Hospital(No. ZM201804) and Beijing Kanghua Foundation for the Development of Traditional Chinese and Western Medicine -Le Fund (KH-2020-LJJ-021, KH-2021-LLZX-058).

## Conflict of Interest

The authors declare that the research was conducted in the absence of any commercial or financial relationships that could be construed as a potential conflict of interest.

## Publisher’s Note

All claims expressed in this article are solely those of the authors and do not necessarily represent those of their affiliated organizations, or those of the publisher, the editors and the reviewers. Any product that may be evaluated in this article, or claim that may be made by its manufacturer, is not guaranteed or endorsed by the publisher.

## Abbreviations

PARP, Poly ADP-ribose polymerase; PARPi, Poly ADP-ribose polymerase inhibitor; BRCAmt, BRCA mutation type; HRD, Homologous recombination deficiency; AEs, Adverse events; NCI CTCAE, National Cancer Institute Common Terminology Criteria for Adverse Events; DCR, Disease control response; PFS, Progression-free survival; SAEs, Serious AEs; SSBs, Single-strand breaks; DSBs, Double strand breaks; HRR, Homologous recombination repair; PrAEs, PARPi-related adverse events; RECIST 1.1, Response Evaluation Criteria in Solid Tumors 1.1; ECOG PS, Eastern Cooperative Oncology Group performance status; CR, Complete response; PR, Partial response; SD, Stable disease; PD, progressive disease; OS, Overall survival.
