# Supplementary material for: Adverse Events as a Potential Clinical Marker of Antitumor Efficacy in Ovarian Cancer Patients Treated With Poly ADP-Ribose Polymerase Inhibitor
Source: Front Oncol. 2021 Sep 6;11:724620. doi: 10.3389/fonc.2021.724620 (PMC8450569; doi:10.3389/fonc.2021.724620)
Supplement: Supplementary file 1 [file Table_1.doc]

**Table S1** Baseline characteristics of 78 patients. Values are reported as frequency (n [%]) or as mean (range)

| Characteristic | Number of patients (percent) |
| --- | --- |
| Age, years |  |
| Median age (range) | 56 (30-80) |
| ≤55 | 35 (44.9) |
| >55 | 43 (55.1) |
| ECOG PS |  |
| 0 | 42 (53.8) |
| 1 | 36 (46.2) |
| Primary tumor location |  |
| Ovary | 74 (94.9) |
| Fallopian tube | 4 (5.1) |
| International FIGO stage |  |
| I-Ⅱ | 10 (12.8) |
| Ⅲ-Ⅳ | 65 (83.3) |
| Unknown | 3 (3.8) |
| Histological type |  |
| Serous | 69 (88.5) |
| Endometrioid | 4 (5.1) |
| Mixed | 1 (1.3) |
| Carcinosarcoma | 1 (1.3) |
| Other | 1 (1.3) |
| Unknown | 2 (2.6) |
| Family history of cancer |  |
| Yes | 15 (19.2) |
| No | 29 (37.2) |
| Unknown | 34 (43.6) |
| Platinum sensitive |  |
| Yes | 35 (44.9) |
| No | 14 (17.9) |
| Unknown | 29 (37.2) |
| Neoadjuvant chemotherapy |  |
| Yes | 15 (19.2) |
| No | 54 (69.2) |
| Unknown | 9 (11.5) |
| PDS/IDS |  |
| Yes | 74 (94.9) |
| No | 4 (5.1) |
| Secondary cytoreductive surgery |  |
| Yes | 16 (20.5) |
| No | 58 (74.4) |
| Unknown | 4 (5.1) |
| Prior lines of chemotherapy |  |
| <3 | 35 (44.9) |
| ≥3 | 43 (55.1) |
| HRD status |  |
| Positive | 36 (46.2) |
| Negative | 14 (17.9) |
| Unknown | 28 (35.9) |
| Residual disease at PDS/IDS |  |
| R0 | 43 (55.1) |
| 1 cm or less | 31 (39.7) |
| Greater than 1 cm | 0 (0) |
| Unknown | 4 (5.1) |
| Categories of PARP inhibitors |  |
| Olaparib | 48 (61.5) |
| Niraparib | 30 (38.5) |

Abbreviations: ECOG PS, Eastern Cooperative Oncology Group performance status; FIGO, International Federation of Gynecology and Obstetrics; HRD, homologous recombination deficiency; PDS, primary debulking surgery; IDS, interval debulking surgery; R0, no macroscopic disease.

**Table S2** Disease control rate of Olaparib-related anemia and Niraparib-related thrombocytopenia.

| AEs | Disease control rate  (%) | $\chi^{2}$ | *P*-value |
| --- | --- | --- | --- |
| Olaparib |  |  |  |
| Anemia |  | 7.403 | 0.007 |
| Yes | 83.78 |  |  |
| No | 36.36 |  |  |
| Time before anemia occurred |  | 0.133 | 0.715 |
| ≤4 weeks | 85.71 |  |  |
| >4 weeks | 81.25 |  |  |
| Grade of anemia |  | Exact probability test | 0.149 |
| 1-2 | 76.00 |  |  |
| 3-4 | 100.00 |  |  |
| Niraparib |  |  |  |
| Thrombocytopenia |  | 0.209 | 0.647 |
| Yes | 66.67 |  |  |
| No | 81.82 |  |  |
| Time before thrombocytopenia occurred |  | 4.052 | 0.044 |
| ≤4 weeks | 86.67 |  |  |
| >4 weeks | 40.00 |  |  |
| Grade of thrombocytopenia |  | 0.000 | 1.000 |
| 1-2 | 70.59 |  |  |
| 3-4 | 62.50 |  |  |

Abbreviations: AEs, adverse events.

**Table S3** Baseline characteristics of patients with AEs and hematological toxicity occurred in different weeks.

| Baseline characteristics | AEs (≤1 week)  (N=36), N (%) | AEs (>1 week)  (N=41), N (%) | *P*-value | Baseline characteristics | Hematological toxicity  (≤4 weeks)  (N=38), N (%) | Hematological toxicity  (>4 weeks)  (N=22), N (%) | *P*-value |
| --- | --- | --- | --- | --- | --- | --- | --- |
| Age, years |  |  | 0.821 | Age, years |  |  | 0.593 |
| Median age (range) | 56 (30-77) | 56 (39-80) |  | Median age (range) | 55 (30-78) | 56 (41-73) |  |
| ≤55 | 17 (47.2) | 18 (43.9) |  | ≤55 | 21 (55.3) | 10 (45.5) |  |
| >55 | 19 (52.8) | 23 (56.1) |  | >55 | 17 (44.7) | 12 (54.5) |  |
| ECOG PS |  |  | 0.113 | ECOG PS |  |  | 0.175 |
| 0 | 16 (44.4) | 26 (63.4) |  | 0 | 25 (65.8) | 10 (45.5) |  |
| 1 | 20 (55.6) | 15 (36.6) |  | 1 | 13 (34.2) | 12 (54.5) |  |
| Primary tumor location |  |  | 0.044 | Primary tumor location |  |  | 0.619 |
| Ovary | 32 (88.9) | 41 (100.0) |  | Ovary | 36 (94.7) | 20 (90.9) |  |
| Fallopian tube | 4 (11.1) | 0 (0.0) |  | Fallopian tube | 2 (5.3) | 2 (9.1) |  |
| International FIGO stage* |  |  | 0.310 | International FIGO stage* |  |  | 1.000 |
| I-Ⅱ | 3 (8.3) | 7 (18.4) |  | I-Ⅱ | 5 (13.5) | 2 (10.0) |  |
| Ⅲ-Ⅳ | 33 (91.7) | 31 (81.6) |  | Ⅲ-Ⅳ | 32 (86.5) | 18 (90.0) |  |
| Histological type* |  |  | 0.118 | Histological type* |  |  | 0.506 |
| Serous | 34 (97.1) | 34 (85.0) |  | Serous | 31 (86.1) | 22 (100) |  |
| Endometrioid | 0 (0) | 4 (10.0) |  | Endometrioid | 3 (8.3) | 0 (0) |  |
| Mixed | 0 (0) | 1 (2.5) |  | Mixed | 1 (2.8) | 0 (0) |  |
| Carcinosarcoma | 1 (2.9) | 0 (0) |  | Carcinosarcoma | 1 (2.8) | 0 (0) |  |
| Other | 0 (0) | 1 (2.5) |  | Other | 0 (0) | 0 (0) |  |
| Family history of cancer* |  |  | 0.347 | Family history of cancer* |  |  | 0.940 |
| Yes | 6 (27.3) | 9 (42.9) |  | Yes | 6 (30.0) | 4 (36.4) |  |
| No | 16 (72.7) | 12 (57.1) |  | No | 14 (70.0) | 7 (63.6) |  |
| Platinum sensitive* |  |  | 1.000 | Platinum sensitive* |  |  | 0.712 |
| Yes | 16 (72.7) | 19 (70.4) |  | Yes | 18 (72.0) | 13 (81.3) |  |
| No | 6 (27.3) | 8 (29.6) |  | No | 7 (28.0) | 3 (18.8) |  |
| Neoadjuvant chemotherapy* |  |  | 0.563 | Neoadjuvant chemotherapy* |  |  | 1.000 |
| Yes | 6 (18.2) | 9 (25.7) |  | Yes | 7 (21.2) | 5 (23.8) |  |
| No | 27 (81.8) | 26 (74.3) |  | No | 26 (78.8) | 16 (76.2) |  |
| PDS/IDS |  |  | 0.335 | PDS/IDS |  |  | 0.528 |
| Yes | 33 (91.7) | 40 (97.6) |  | Yes | 36 (94.7) | 22 (100) |  |
| No | 3 (8.3) | 1 (2.4) |  | No | 2 (5.3) | 0 (0) |  |
| Secondary cytoreductive surgery* |  |  | 1.000 | Secondary cytoreductive surgery* |  |  | 1.000 |
| Yes | 8 (22.2) | 8 (21.6) |  | Yes | 9 (25.0) | 5 (23.8) |  |
| No | 28 (77.8) | 29 (78.4) |  | No | 27 (75.0) | 16 (76.2) |  |
| Prior lines of chemotherapy |  |  | 0.821 | Prior lines of chemotherapy |  |  | 1.000 |
| <3 | 17 (47.2) | 18 (43.9) |  | <3 | 15 (39.5) | 9 (40.9) |  |
| ≥3 | 19 (52.8) | 23 (56.1) |  | ≥3 | 23 (60.5) | 13 (59.1) |  |
| HRD status* |  |  | 1.000 | HRD status* |  |  | 0.488 |
| Positive | 18 (72.0) | 17(70.8) |  | Positive | 16 (66.7) | 11 (78.6) |  |
| Negative | 7 (28.0) | 7 (29.2) |  | Negative | 8(33.3) | 3 (21.4) |  |
| BRCA status |  |  | 0.567 | BRCA status |  |  | 0.101 |
| Mutation type | 9(36.0) | 12(48.0) |  | Mutation type | 8(34.8) | 9(64.3) |  |
| Wild type | 16(64.0) | 13(52.0) |  | Wild type | 15(65.2) | 5(35.7) |  |
| Residual disease at PDS/IDS* |  |  | 0.354 | Residual disease at PDS/IDS* |  |  | 0.104 |
| R0 | 21 (63.6) | 21 (52.5) |  | R0 | 22 (61.1) | 8 (36.4) |  |
| 1 cm or less | 12 (36.4) | 19 (47.5) |  | 1 cm or less | 14 (38.9) | 14 (63.6) |  |
| Greater than 1 cm | 0 (0) | 0 (0) |  | Greater than 1 cm | 0 (0) | 0 (0) |  |
| Categories of PARP inhibitors |  |  | 0.157 | Categories of PARP inhibitors |  |  | 0.774 |
| Olaparib | 19 (52.8) | 29 (70.7) |  | Olaparib | 25 (65.8) | 16 (72.7) |  |
| Niraparib | 17 (47.2) | 12 (29.3) |  | Niraparib | 13 (34.2) | 6 (27.3) |  |

Abbreviations: ECOG PS, Eastern Cooperative Oncology Group performance status; FIGO, International Federation of Gynecology and Obstetrics; HRD, homologous recombination deficiency; PDS, primary debulking surgery; IDS, interval debulking surgery; R0, no macroscopic disease; *, This characteristic contains unknown (missing/non-observed) data.

**Table S4** Classification of interval validation.

| Observed | | Predicted | | |
| --- | --- | --- | --- | --- |
|  |  | DCR | | Percentage correct (%) |
|  |  | Disease progression | Disease control |  |
| DCR | Disease progression | 6 | 15 | 28.6 |
|  | Disease control | 2 | 49 | 96.1 |
| Overall percentage | |  |  | 76.4 |

Abbreviations: DCR, disease control rate.
